# Screening and HPLC-Based Activity Profiling for New Antiprotozoal Leads from European Plants

**DOI:** 10.3797/scipharm.1111-13

**Published:** 2011-12-23

**Authors:** Stefanie Zimmermann, Semira Thomi, Marcel Kaiser, Matthias Hamburger, Michael Adams

**Affiliations:** 1 Departement of Pharmaceutical Sciences, Pharmaceutical Biology, University of Basel, Klingelbergstrasse 50, 4056, Basel, Switzerland; 2 Parasite Chemotherapy, Department of Medical Parasitology and Infection Biology, Swiss Tropical and Public Health Institute, Socinstrasse 57, 4002 Basel, Switzerland

**Keywords:** European plants, *Plasmodium falciparum*, *Trypanosoma brucei rhodesiense*, *Arctium nemorosum*, Onopordopicrin

## Abstract

Based on a survey of remedies used in Renaissance Europe to treat malaria, we prepared and screened a library of 254 extracts from 61 plants for antiplasmodial activity *in vitro*. HPLC-based activity profiling was performed for targeted identification of active constituents in extracts. One of the most remarkable results was the identification of onopordopicrin, a germacranolide sesquiterpene lactone isolated from *Arctium nemorosum* as a potent inhibitor of *P. falciparum* with an IC_50_ of 6.9 μM. It was tested similarly against *Trypanosoma brucei rhodesiense*, the parasite which causes African sleeping sickness. With an IC_50_ of 0.37 μM, onopordopicrin was one of the most potent natural products reported so far. Cytotoxicity was determined against rat myoblast L6 cells (IC_50_: 3.06).

## Introduction

Malaria is still the most deadly parasitic disease in the world, leading to one million deaths every year, mostly in Sub-Saharan Africa. In 1976 Trager and Jensen [1] developed a method for propagation of *Plasmodium falciparum* in human erythrocytes. This parasite species is the cause of malaria tropica, the most severe form of malaria in humans [2]. This assay opened up the possibility of screening large numbers of samples for antiplasmodial activity. One particular source of samples which deserves special attention is plants that local populations use to treat malaria in regions where it is endemic. This is a reasonable approach to lead discovery considering that nowadays *P. falciparum* malaria is treated with artemisinin-based combination therapies (ACT) such as artemether/lumefantrine, artesunate/amodiaquine, artesunate/mefloquine and artesunate/sulfadoxine-pyrimethamine [3]. Artemisinin is a natural compound isolated from the medicinal herb *Artemisia annua* L. (Asteraceae) which was used in China to treat fever including malaria. Combination of artemisinin derivatives with other antimalarial drugs is necessary, firstly to increase efficacy and secondly to prevent resistances [3].

More than 1200 plant species from 160 families have been described as traditional antimalarial remedies, and hundreds of extracts and purified compounds from such plants have shown antiplasmodial activities tested in the last decades [4]. Bero et al [5] recently reviewed more than 300 antiplasmodial (IC_50_s < 11 μM) plant compounds isolated from traditionally used plants, all of which had been published in the three years from 2005 through 2008. Among them were 31 compounds with IC_50_ below 2 μM, which were termed “promising” compounds [5]. They argued that “ethnopharmacological approaches appear to be a promising way to find plant metabolites that could be used as templates for designing new derivatives with improved properties". Willcox recently reviewed clinical studies including 18 case studies and 39 cohort studies on traditional herbal malaria treatments [6].

Malaria is nowadays mostly seen as a tropical disease, and it is common in tropical countries where most indigenous knowledge and plants are collected. Yet, all through history and until the 20^th^ century malaria caused by *P. vivax* and *P. malariae* was widespread in Europe [7]. We recently did a search in eight herbals in German language from the 16^th^ and 17^th^ centuries and identified 314 plants that had been used to treat what was then known as tertian (*P. vivax*) and quartan (*P. malariae*) fever. However, one finding from this study was that only five percent of these plants had ever been studied for antiplasmodial effects and only one had been assayed *in vivo* [8].

Considering the apparent lack of exploitation of this knowledge, we set out for a screening involving plants identified in our survey. We purchased or collected 61 European medicinal plants from various sources (see Table S1 in the Supporting Information), 34 of which had been described as antimalarials in the herbals [8]. The other 27 were taken as a control group, as they too were commercial medicinal plants or plants from genera which had medicinal uses. The plants were dried, separated in different parts (roots, aerial parts, flowers, etc) and extracted successively with *n*-hexane, ethyl acetate, and methanol to give three extracts of increasing polarity for each sample. This focused extract library was then tested for *in vitro* inhibition of *P. falciparum*. Follow-up of active extracts was by HPLC-based activity profiling to identify the active constituents [9]. We recently adapted and validated this approach for the miniaturized and efficient identification of antiprotozoal compounds in complex plant and fungal extracts [10] and have successfully applied it to discovery of new antiprotozoal natural products in other library-based discovery projects [11–17].

## Results and Discussion

Two hundred and fifty-four extracts were prepared from 61 plants and tested for antiplasmodial activity at two test concentrations (0.8 and 4.8 μg/ml) according to an established procedure. The identity of the plants, their botanical authority, their origin, the plant part used, the extraction solvent and the % inhibition with the standard deviation as the mean of the three repetitions are all shown in Table S1.

Nine of the 10 most active extracts in this screen (at 4.8 μg/ml) were from plants with a documented antimalarial use in Renaissance herbals [8] (Table S1). These included the petroleum ether extract of the aerial parts of *Peucedanum ostruthium* (L.) Koch (71% inhibition), the ethyl acetate extract of the roots of *Asparagus officinalis* L. (75%), the ethyl acetate extract of the aerial parts of *Artemisia abrotanum* L. (69%), the petroleum ether extract of the leaves of *Artemisia absinthium* L. (68%), the ethyl acetate extract of the roots of *Inula conyzae* (Griess.) Meikle (66%), the ethyl acetate extract of the flowers of *Humulus lupulus* L. (96%), the ethyl acetate extract of the roots of *Anacyclus pyrethrum* (L.) Link (65%), the ethyl acetate extract of the aerial parts of *Hypericum perforatum* L. (98%), and the petroleum ether extract of the aerial parts of *Hyssopus officinalis* L. (66%). Finally, the ethyl acetate extract of the leaves of *Arctium nemorosum* Lej., which had no record of antimalarial use, was particularly effective (99%).

Some of the active plants, including *Peucedanum ostruthium*, *Asparagus officinalis, Humulus lupulus, Artemisia absinthium* and *Hypericum perforatum* had previously been studied for antiplasmodial effects by other groups (see [8]) and were therefore not followed up in this study. We decided to proceed with HPLC-based activity profiling with the active extracts from *Arctium nemorosum* and *Hyssopus officinalis* to determine their active constituents. The lack of previous phytochemical studies of *A. nemorosum* made this plant species in particular interesting to us.

### Hyssopus officinalis L

**Fig. 1:**
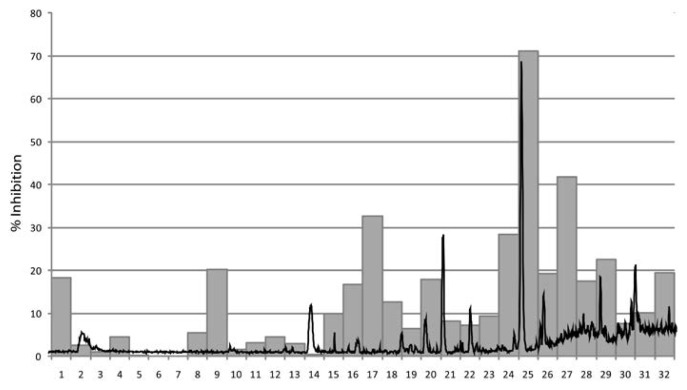
Activitiy profiling of *Hyssopus officinalis*. The antiplasmodial activity of the 32 one-minute microfractions is plotted against the mass trace of the petrol ether extract (ESI-ESI positive scan *m/z* 150–1500).

The HPLC-based activity profile of the *H. officinalis* extract showed that the activity was concentrated in one major time window at minute 26, which also contained the most dominant peak in the chromatogram (Fig. 1).

HR-HPLC-MS (method shown in the Supporting Information) analysis of the peak with the retention time 25.3 minutes showed m/*z* 293.4263 [M-H]^−^, which was indicative of a molecular formula of C_18_H_30_O_3_ (calc. for 294.4290).

**Fig. 2:**
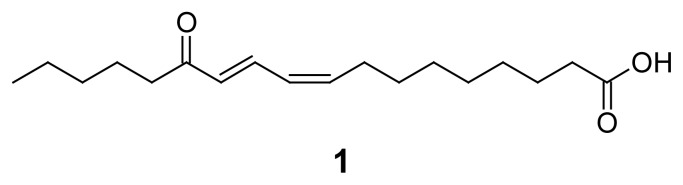
13-Oxo-9Z, 11E-octadecadienoic acid

The compound corresponding to this peak was then isolated by the following procedure: Two kilograms of dried and finely ground aerial parts of *Hyssopus officinalis* (Dixa AG Herbs and Spices, St. Gallen, Switzerland) were extracted three times with eight L of *n*-heptane, which yielded 19 g of a dark viscous extract. MPLC was done to separate 5 g extract into 129 fractions (Method 1). Fractions were analysed by HPLC-ESI-MS and TLC (see Supporting Information) after every separation step to identify fractions containing targeted compound **1**. Fractions F_80–85_ were pooled and preparative HPLC (see Supporting information) was applied to isolate 0.5 mg of **1**. By comprehensive use of HR-MS, 1D and 2D NMR, and comparison with literature [17], **1** was identified as 13-oxo-9*Z*,11*E*-octadeca-dienoic acid (**1**, Fig. 2). This compound was previously reported from *H. officinalis* [18].

This prompted us to test a series of saturated and unsaturated fatty acids for their antiplasmodial effects alongside **1**. These compounds were 10-cis-heptadecenoic acid, 11-cis-eicosenoic acid, 13-cis-docosenoic acid, methyl linoleate, linoleic acid, caproic acid, docosanoic acid, arachidic acid, 9,12,15-all-cis-octadecaterienic acid, and caprylic acid. Neither **1** nor any of these fatty acids had more than 50% inhibition at a test concentration of 10 μg/mL.

### Arctium nemorosum Leij

**Fig. 4:**
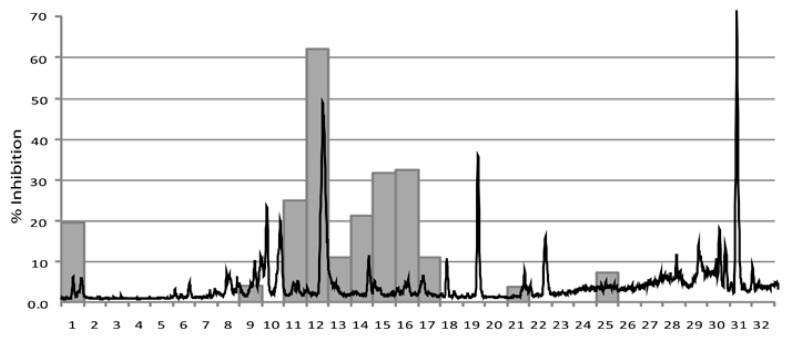
Activity profiling of *Arctium nemorosum*. The antiplasmodial activity of the 32 one-minute microfractions is plotted against the mass trace of the ethyl acetate extract (ESI-ESI positive scan *m/z* 150–1500).

The HPLC-based activity profile of the *A. nemorosum* extract showed that the major peak of activity was concentrated in one major time window at minute 12. This time window also contained the most dominant peak in the chromatogram (Fig. 4). HR-HPLC-MS analysis (method in the Supporting Information) of the peak with retention time 12.6 min showed m/*z* 719.3088 [2M+Na]^+^, which was indicative of a molecular formula of C_29_H_24_O_6_ (calc. for 348.1580).

**Fig. 5:**
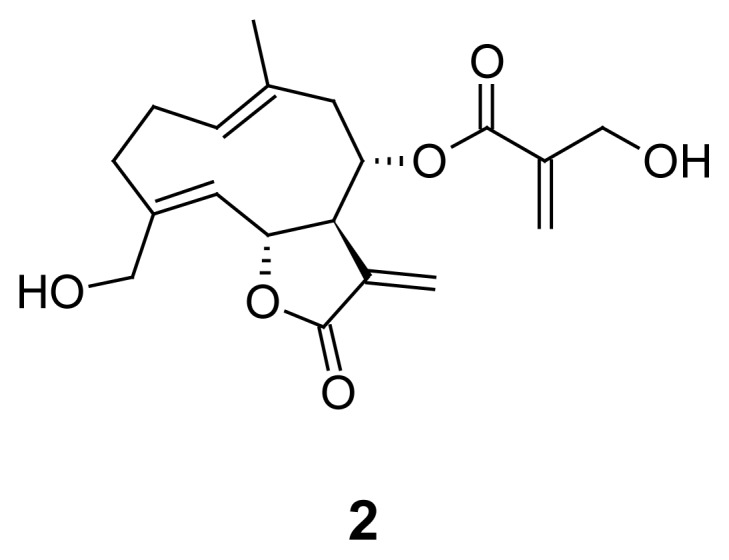
Onopodopicrin

The aerial parts of *A. nemorosum* (28.6 g) were milled and extracted exhaustively with EtOAc to afford 1.16 g of dried extract. The active peak at RT 12.5 seen in the HPLC-based activity profiling (Fig. 4) was isolated by semi-preparative HPLC (Method 1) yielding 5.71 mg. The structure was established by comprehensive analysis of HR-MS and 2D NMR data as the germacranolide sesquiterpene lactone onopordopicrin (**2**, Fig. 5). NMR and MS data was in accordance with literature [19]. Onopordopicrin has been previously reported as constituent in leaves of *Onopordum acanthium* L. and *Arctium lappa* L. [19, 20].

Onopodopicrin (**2**) was an effective inhibitor of *P. falciparum* parasite growth, with an IC_50_ of 6.89 ± 0.56 μM. The compound shares some structural features, such as the lactone ring with an exocyclic methylene group, and a 2-(hydroxymethyl) acrylate side chain, with cynaropicrin, which we recently identified as an *in vivo* active compound in the acute *T. brucei rhodesiense* sleeping sickness mouse model [16]. Therefore, we tested **2** against *T. brucei rhodesiense in vitro* as well. Onopordopicrin (**2**) showed potent activity with an IC_50_ value of 0.37 ± 0.01 μM. Against rat myoblast cells (L6 cells) it was cytotoxic with an IC_50_ of 3.06 ± 1.1 μM, and thus a selectivity index (SI) of 8.3.

In summary, 254 extracts from 61 medicinal plants (Table S1) were prepared and screened for antiplasmodial effects. Thirty-four of the plants had been described as antimalarials in German Renaissance herbals [8] and 27 were regarded as a control group. Amongst the most potent 10 extracts, nine were from those plants which had had an antimalarial use. *Arctium nemorosum,* a plant which was very active, was not reported in the herbals [8]. However, the closely related species *Arctium lappa* had been used as a traditional malaria remedy (Table S1). The extracts of *A. lappa* were included in this screening, too, and showed slightly lower activity against *P. falciparum* than *A. nemorosum* (Table S1). HPLC-based activity profiling was done with two of the active extracts, namely the petroleum ether extract of *Hyssopus officinalis* and the ethyl acetate extract of *Arctium nemorosum*. In both cases the active compounds were rapidly identified and isolated. In the case of *H. officinalis* the isolated compound was 13-oxo-9*Z*, 11*E*-octadecadienoic acid, an unusual keto fatty acid. Tested as a pure compound its IC_50_ was higher than the highest test concentration of 10 μg/ml. A possible explanation for this may be that due to its high concentration in the extract, the compound nevertheless showed up in the activity profile (Fig. 1). Thus, the presence of “false positives”, that is, major and with weak activity must be considered when applying these approaches. Also, it may be that another compound we could not detect was present in this time window and was responsible for the observed activity.

In the case of *Arctium nemorosum* onopordopicrin (**2**) was identified as being chiefly responsible for the observed activity. Its *in vitro* antiplasmodial (*P. falciparum* IC_50_= 6.89 ± 0.56) and antitrypanosomal (*T. b. rhodesiense* IC_50_= 0.37 ± 0.01) effects were similar to those of cynaropicrin (*P. falciparum* IC_50_= 3.00 ± 0.28; *T. b. rhodesiense* IC_50_= 0.28 ± 001, L6-cells IC_50_= 2.19 ± 0.27), which has previously been shown to possess *in vivo* antitrypanosomal activity [17]. HPLC-based activity profiling is thus a fast and efficient method for identifying active compounds in complex extracts [9, 10]. The selection of traditionally used plants for a focused screening as a rational approach to lead discovery is supported here.

## Experimental

Analytical grade solvents for extraction and HPLC grade solvents for chromatography were purchased from Scharlau (Barcelona, Spain). HPLC grade water was obtained by an EASY-pure II (Barnstead; Dubuque IA, USA) water purification system.

### Plant material

Plant material was purchased from commercial sources or collected by the author (M. Adams), and voucher specimens were deposited at the Department of Pharmaceutical Sciences, University of Basel. Origins and voucher numbers are shown in the Supporting Information.

### Extraction of plant material for screening

Plants were dried and separated into different parts (roots, aerial parts, flowers) and finely ground using a ZM1 ultra centrifugal mill (Retsch; Haan, Germany). 1 g of powdered material was then successively extracted first with petroleum ether, then ethyl acetate and finally methanol using an accelerated solvent extraction system ASE (ASE 200, Dionex, Switzerland; 3 cycles at 120 bar, and 70°C) to give a set of three extracts of increasing polarity for every sample. The extracts were formatted into a library of solutions at 10 mg/ml DMSO in 2D-barcoded 96 well plates (Zinsser Analytics, France) and stored at −80° C, until used for screening and for HPLC-based activity profiling.

### Screening of extracts for antiplasmodial activity

The antiplasmodial screening was performed as previously described [8]. Tests were repeated three times in duplicate, at test concentrations of 0.8 and 4.8 μg/ml.

### HPLC-based activity profiling

Selected extracts were separated by analytical HPLC for activity profiling as previously reported [10], with the following modifications: extracts were separated by HPLC into 96-deep well plates (ScreenMates 96 well, Matrix Technology, Hudson, USA) and dried within 2 h in a parallel evaporator (EVX-96 Apricot Evaporex, Monrovia, USA), with an N_2_-stream (60 °C upper part/40°C lower part). The dried microfractions were dissolved in 50 μl methanol and transferred into 96 well conical plates (V-well plates, Thermo Scientific, USA). After rinsing with 50 μl MeOH, the plate was again dried using the same condition as described above. The dried microfractions were dissolved in 5 μL of DMSO and then diluted with 95 μL of PBS buffer (137 mM NaCl, 10 mM phosphate, 2.7 mM KCl, pH 7.4). This gave the stock solutions which could be used for the assays [10]. On-line collection of mass spectral data was done by LC ion trap ESI-MS and HR-TOF-MS [10]. Details are given in the Supporting Information.

### Bioassays

Screening of extracts, HPLC-based activity profiling and pure compounds were tested as previously described [10, 17]. Tests were done in three independent assays in duplicate. For details please refer to the Supporting Information.

## Supporting Information



## Figures and Tables

**Fig. 3:**
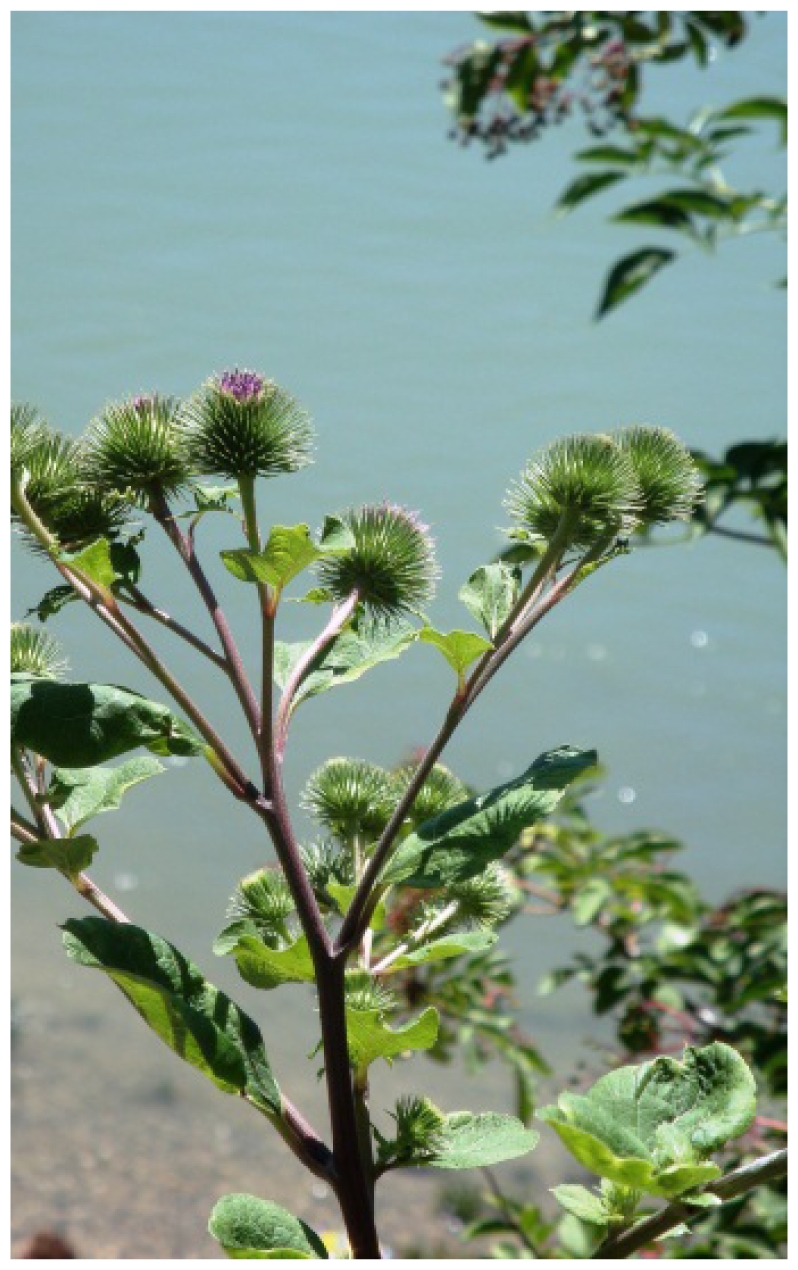
*Arctium nemorosum* Leij., May 2011 (by S. Zimmermann), The river Rhine in the background.
