# Supplementary material for: Screening and HPLC-Based Activity Profiling for New Antiprotozoal Leads from European Plants
Source: Sci Pharm. 2011 Dec 23;80(1):205–13. doi: 10.3797/scipharm.1111-13 (PMC3293357; doi:10.3797/scipharm.1111-13)
Supplement: Supplementary file 1 [file Scipharm-2012-80-205_supporting_information.pdf]

Scientia Pharmaceutica www.scipharm.at

Open Access

Supporting Information to

Screening and HPLC-Based Activity Profiling for New Antiprotozoal Leads from European Plants

Stefanie ZIMMERMANN, Semira THOMI, Marcel KAISER, Matthias HAMBURGER, Michael ADAMS

Published in Sci Pharm. 2012; 80: 205–213 doi:10.3797/scipharm.1111-13

Available from: http://dx.doi.org/10.3797/scipharm.1111-13

© Zimmermann *et al.*; licensee Österreichische Apotheker-Verlagsgesellschaft m. b. H., Vienna, Austria.

This is an Open Access article distributed under the terms of the Creative Commons Attribution License (http://creativecommons.org/licenses/by/3.0/), which permits unrestricted use, distribution, and reproduction in any medium, provided the original work is properly cited.

Table of Contents

Tab. S1. Antiplasmodial activity (growth inhibition in % ± standard deviation [SD]) of 254 plant extracts against Plasmodium falciparum. Bioassays were carried out in duplicate of three independent experiments, at test concentrations of 4.81 µg/mL and 0.81 µg/mL, respectively. The positive control was artesunate (100% inhibition in all bioassays).

Analytical Methods

Tab. S1. Antiplasmodial activity (growth inhibition in % ± standard deviation [SD]) of 254 plant extracts against Plasmodium falciparum. Bioassays were carried out in duplicate of three independent experiments, at test concentrations of 4.81 µg/mL and 0.81 µg/mL, respectively. The positive control was artesunate (100% inhibition in all bioassays).

Plant Family	Plant species	Historical source	Src.	Voucher specimen	Plant part	extract solvent	growth inhib. at 4.81 µg/mL ± SD[a]	growth inhib. at 0.81 µg/mL ± SD[a]
Adoxaceae	*Sambucus ebulus* L.	Br., Lo., Ta.2	A	P01663	leaves	PE	12.3 ± 6.9	1.9 ± 1.4
				P01664	leaves	EtOAc	9.1 ± 12.2	1.5 ± 2.1
				P01665	leaves	MeOH	3.3 ± 4.7	1.3 ± 1.8
				P01669	fruits	PE	20.5 ± 15.0	0.2 ± 0.2
				P01670	fruits	EtOAc	15.8 ± 15.2	8.8 ± 11.8
				P01671	fruits	MeOH	15.9 ± 20.3	2.7 ± 3.8
			A	P01547	roots	PE	58.0 ± 10.3	6.8 ± 5.2
				P01548	roots	EtOAc	9.4 ± 5.1	5.4 ± 5.2
				P01549	roots	MeOH	29.0 ± 9.0	9.7 ± 7.5
	Sambucus nigra L.	Lo., Ta.2	A	P01681	flowers	PE	7.8 ± 6.6	2.2 ± 1.9
				P01682	flowers	EtOAc	7.5 ± 5.8	0.0 ± 0.0
				P01683	flowers	MeOH	9.9 ± 7.1	6.5 ± 4.8
Amaryllidaceae	*Allium ursinum* L.	–	A	P01592	aer. pts.	PE	7.9 ± 7.6	0.0 ± 0.0
				P01593	aer. pts.	EtOAc	5.5 ± 4.0	0.6 ± 0.9
				P01594	aer. pts.	MeOH	13.4 ± 5.9	1.1 ± 2.0
Apiaceae	*Angelica archangelica* L.	Ta.2	A	P01610	fruits	PE	16.0 ± 9.6	10.9 ± 5.7
				P01611	fruits	EtOAc	15.6 ± 11.7	14.5 ± 20.5
				P01612	gruits	MeOH	17.2 ± 8.2	8.6 ± 7.4
				P01613	fer. pts.	PE	32.9 ± 20.2	28.4 ± 24.2
				P01614	aer. pts.	EtOAc	35.0 ± 8.7	8.4 ± 6.8
				P01615	aer. pts.	MeOH	9.9 ± 7.1	7.7 ± 5.5
				P01616	roots	PE	36.6 ± 16.0	29.1 ± 41.1
				P01617	roots	EtOAc	41.6 ± 3.5	19.4 ± 6.1
				P01618	roots	MeOH	17.6 ± 1.8	12.7 ± 9.0
	Angelica sylvestris L.	Ta.2	B	SZ0001	fruits	PE	34.0 ± 5.0	5.2 ± 3.8
				SZ0002	fruits	EtOAc	51.0 ± 6.6	1.7 ± 2.5
				SZ0003	fruits	MeOH	4.9 ± 5.4	1.5 ± 1.1
				SZ0004	leaves	PE	6.7 ± 0.7	0.0 ± 0.0
				SZ0005	leaves	EtOAc	54.6 ± 3.0	4.4 ± 3.5
				SZ0006	leaves	MeOH	17.5 ± 23.1	5.0 ± 3.9
				SZ0007	roots	PE	35.6 ± 2.3	10.2 ± 2.6
				SZ0008	roots	EtOAc	52.3 ± 1.3	8.6 ± 6.1
				SZ0009	roots	MeOH	16.5 ± 2.5	2.3 ± 2.1
	Coriandrum sativum L.	Br., Ta.2	A	P01678	seed	PE	6.3 ± 9.0	2.9 ± 4.1
				P01679	seed	EtOAc	3.3 ± 1.0	7.8 ± 11.1
				P01680	seed	MeOH	6.9 ± 6.7	0.0 ± 0.0
	Eryngium campestre L.	Ma., Ta.1, Ta.2, Zw.	A	P01559	roots	PE	36.9 ± 11.7	4.8 ± 1.9
				P01560	roots	EtOAc	44.5 ± 6.5	0.0 ± 0.0
				P01561	roots	MeOH	13.6 ± 3.2	9.9 ± 5.5
				P01565	aer. pts.	PE	64.1 ± 2.3	1.6 ± 4.0
				P01566	aer. pts.	EtOAc	16.9 ± 5.5	0.1 ± 4.4
				P01567	aer. pts.	MeOH	34.4 ± 3.8	0.0 ± 0.0
Apiaceae	*Foeniculum vulgare* Mill. subsp. *vulgare* var. *dulce* (Mill.)	Ta.2	A	P01577	aer. pts.	PE	29.7 ± 2.2	2.5 ± 1.8
				P01578	aer. pts.	EtOAc	8.7 ± 3.5	0.0 ± 0.0
				P01579	aer. pts.	MeOH	5.1 ± 2.5	1.5 ± 2.1
	Peucedanum ostruthium (L.) Koch	Bo., Br., Ma., Zw.	A	P01587	rhizome	PE	11.7 ± 8.0	0.0 ± 0.0
				P01588	rhizome	EtOAc	16.4 ± 4.2	1.7 ± 2.4
				P01586	rhizome	MeOH	15.4 ± 9.8	0.0 ± 0.0
				P01590	aer. pts.	PE	71.1 ± 8.1	0.0 ± 0.0
				P01591	aer. pts.	EtOAc	7.8 ± 6.7	3.9 ± 5.5
				P01589	aer. pts.	MeOH	31.2 ± 3.2	1.2 ± 1.7
Asparagaceae	*Asparagus officinalis* L.	Ta.2, Zw.	A	P01463	roots	PE	26.4 ± 10.9	0.3 ± 0.3
				P01464	roots	EtOAc	75.5 ± 7.5	4.0 ± 4.8
				P01465	roots	MeOH	37.5 ± 0.8	1.6 ± 1.1
Aspleniaceae	*Asplenium scolopendrium* L.	Bo., Lo, Zw.	B	P01666	leaves	PE	4.7 ± 5.9	0.0 ± 0.0
				P01667	leaves	EtOAc	7.4 ± 5.3	0.0 ± 0.0
				P01668	leaves	MeOH	11.3 ± 14.6	0.0 ± 0.0
Asteraceae	*Achillea millefolium* L.	Ta.2	A	P01599	aer. pts.	PE	48.4 ± 4.1	0.0 ± 0.0
				P01600	aer. pts.	EtOAc	9.3 ± 1.1	0.0 ± 0.0
				P01598	aer. pts.	MeOH	16.6 ± 7.1	0.5 ± 0.3
	Achillea moschata Wulfen	–	A	P01499	aer. pts.	PE	42.6 ± 17.2	5.9 ± 5.0
				P01500	aer. pts.	EtOAc	36.5 ± 12.6	0.0 ± 0.0
				P01501	aer. pts.	MeOH	8.4 ± 7.2	0.0 ± 0.0
	Anacyclus pyrethrum (L.)	Bo., Ma., Ta.2, Zw.	B	P01637	roots	PE	15.0 ± 6.1	0.4 ± 0.6
				P01638	roots	EtOAc	65.3 ± 2.8	26.2 ± 32.0

Tab. S1. (Cont).

Plant Family	Plant species	Historical source	Src.	Voucher specimen	Plant part	extract solvent	growth inhib. at 4.81 µg/mL ± SD[a]	growth inhib. at 0.81 µg/mL ± SD[a]
Asteraceae	*Arctium lappa* L.	Zw.	A	P01472	leaves	PE	17.4 ± 1.5	2.0 ± 2.9
				P01473	leaves	EtOAc	33.3 ± 25.7	0.0 ± 0.0
				P01474	leaves	MeOH	8.9 ± 8.1	2.3 ± 3.2
				P01475	roots	PE	50.5 ± 3.0	54.7 ± 2.8
				P01476	roots	EtOAc	23.3 ± 4.0	11.2 ± 2.0
				P01477	roots	MeOH	4.1 ± 3.0	0.3 ± 0.5
	Arctium nemorosum Lej.	–	B	SZ0010	aer. pts	MeOH	53.6 ± 24.8	21.8 ± 29.1
				SZ0011	leaves	PE	16.4 ± 3.9	12.8 ± 2.9
				SZ0012	leaves	EtOAc	99.1 ± 0.4	14.1 ± 10.0
				SZ0013	leaves	MeOH	16.5 ± 3.2	4.3 ± 3.2
				SZ0014	fruits	PE	5.3 ± 3.8	2.3 ± 3.3
				SZ0015	fruits	EtOAc	55.6 ± 4.8	3.1 ± 4.2
				SZ0016	fruits	MeOH	5.8 ± 3.5	0.0 ± 0.0
				SZ0017	hollow stem	PE	2.7 ± 2.0	1.7 ± 2.4
				SZ0018	hollow stem	EtOAc	31.3 ± 5.6	1.3 ± 1.8
				SZ0019	hollow stem	MeOH	57.5 ± 3.4	17.5 ± 3.9
				SZ0020	roots	PE	5.0 ± 4.9	1.7 ± 2.1
				SZ0021	roots	EtOAc	31.0 ± 5.6	0.6 ± 0.8
				SZ0022	roots	MeOH	3.2 ± 4.5	0.0 ± 0.0
	Arnica montana L	–	B	P01451	flowers	PE	18.2 ± 3.8	6.4 ± 4.7
				P01452	flowers	EtOAc	30.7 ± 6.8	4.0 ± 5.7
				P01453	flowers	MeOH	5.7 ± 4.4	0.0 ± 0.0
				P01454	roots	PE	23.1 ± 0.9	14.1 ± 1.3
				P01455	roots	EtOAc	36.1 ± 3.2	5.9 ± 8.3
				P01456	roots	MeOH	13.5 ± 8.0	5.6 ± 4.0
	Artemisia abrotanum L.	Bo., Ma., Ta.2	A	P01433	aer. pts.	PE	50.2 ± 2.4	23.5 ± 8.5
				P01434	aer. pts.	EtOAc	69.3 ± 3.4	18.3 ± 7.9
				P01435	aer. pts.	MeOH	17.8 ± 11.2	8.5 ± 6.0
	Artemisia absinthium L.	Ma., Ta.2, Zw.	B	P01672	leaves	PE	67.9 ± 11.2	5.8 ± 3.8
				P01673	leaves	EtOAc	55.7 ± 14.5	3.8 ± 4.9
				P01674	leaves	MeOH	6.3 ± 7.4	1.0 ± 1.5
	Artemisia dracunculus L.	–	A	P01545	aer. pts.	PE	16.5 ± 3.2	0.5 ± 0.7
				P01546	aer. pts.	EtOAc	4.3 ± 3.0	7.1 ± 5.0
				P01544	aer. pts.	MeOH	24.5 ± 3.2	1.7 ± 1.8
Asteraceae	*Artemisia vulgaris* L.	Ta.2	A	P01457	aer. pts.	PE	18.9 ± 7.0	6.3 ± 2.7
				P01458	aer. pts.	EtOAc	41.9 ± 11.6	0.0 ± 0.0
				P01459	aer. pts.	MeOH	0.0 ± 0.0	0.0 ± 0.0
				P01460	roots	PE	16.5 ± 5.9	5.2 ± 4.1
				P01461	roots	EtOAc	44.1 ± 9.7	5.0 ± 4.3
				P01462	roots	MeOH	17.3 ± 3.8	6.6 ± 5.7
			B	P01645	aer. pts.	PE	31.7 ± 3.4	15.2 ± 11.4
				P01646	aer. pts.	EtOAc	19.4 ± 10.7	0.4 ± 0.7
				P01647	aer. pts.	MeOH	6.0 ± 1.9	0.0 ± 0.0
	Carthamus tinctorius L.	–	A	P01502	flowers	PE	9.1 ± 9.6	1.2 ± 1.7
				P01503	flowers	EtOAc	10.3 ± 2.1	0.4 ± 0.6
				P01504	flowers	MeOH	9.0 ± 6.7	1.0 ± 1.5
	Centaurea cyanus L.	Bo., Lo.	A	P01527	flowers	PE	54.8 ± 9.1	22.3 ± 3.5
				P01528	flowers	EtOAc	19.2 ± 8.0	9.6 ± 6.3
				P01526	flowers	MeOH	28.0 ± 6.6	26.4 ± 7.5
	Centaurea montana L.	–	B	P01648	flowers	PE	40.7 ± 5.8	0.0 ± 0.0
				P01649	flowers	EtOAc	16.6 ± 5.3	18.2 ± 25.7
				P01650	flowers	MeOH	16.5 ± 2.6	0.0 ± 0.1
				P01651	leaves	PE	25.3 ± 8.7	6.5 ± 3.8
				P01652	leaves	EtOAc	10.0 ± 4.6	0.0 ± 0.0
				P01653	leaves	MeOH	8.4 ± 2.7	1.1 ± 1.1
	Cichorium intybus L.	Bo., Ta.2, Zw.	A	P01514	roots	PE	6.0 ± 4.4	0.0 ± 0.0
				P01515	roots	EtOAc	14.2 ± 1.9	2.0 ± 1.4
				P01516	roots	MeOH	1.8 ± 2.3	0.0 ± 0.0
				P01517	aer. pts.	PE	9.5 ± 1.9	0.0 ± 0.0
				P01518	aer. pts.	EtOAc	18.2 ± 11.4	0.1 ± 0.2
				P01519	aer. pts.	MeOH	6.5 ± 6.6	0.0 ± 0.0
			B	P01657	aer. pts.	PE	9.7 ± 8.6	0.0 ± 0.0
				P01658	aer. pts.	EtOAc	9.7 ± 13.7	0.0 ± 0.0
				P01659	aer. pts.	MeOH	3.5 ± 3.3	0.6 ± 0.8
				P01660	roots	PE	15.0 ± 12.3	4.5 ± 6.3
				P01661	roots	EtOAc	21.0 ± 3.1	1.1 ± 1.0
				P01662	roots	MeOH	12.1 ± 3.6	3.9 ± 3.7

Tab. S1. (Cont).

Plant Family	Plant species	Historical source	Src.	Voucher specimen	Plant part	extract solvent	growth inhib. at 4.81 µg/mL ± SD[a]	growth inhib. at 0.81 µg/mL ± SD[a]
Asteraceae	*Echinacea angustifolia* DC.	–	A	P01551	roots	PE	20.6 ± 6.9	4.2 ± 4.0
				P01552	roots	EtOAc	8.9 ± 6.1	1.3 ± 1.9
				P01550	roots	MeOH	12.2 ± 7.8	3.1 ± 3.1
	Echinacea purpurea (L.) Moench	–	A	P01554	roots	PE	29.2 ± 12.8	0.0 ± 0.0
				P01555	roots	EtOAc	19.0 ± 7.4	0.3 ± 0.2
				P01553	roots	MeOH	24.1 ± 9.0	0.0 ± 0.0
				P01557	aer. pts.	PE	23.3 ± 6.5	0.0 ± 0.0
				P01558	aer. pts.	EtOAc	21.9 ± 2.9	10.1 ± 5.2
				P01556	aer. pts.	MeOH	16.2 ± 0.6	1.4 ± 2.0
	Eupatorium cannabinum L.	Ma.	A	P01569	aer. pts.	PE	38.7 ± 9.6	5.2 ± 4.1
				P01570	aer. pts.	EtOAc	11.2 ± 9.1	0.9 ± 0.6
				P01568	aer. pts.	MeOH	17.4 ± 4.0	0.0 ± 0.0
				P01572	aer. pts.	PE	31.6 ± 3.2	5.6 ± 5.3
				P01573	aer. pts.	EtOAc	8.7 ± 1.9	0.0 ± 0.0
				P01571	aer. pts.	MeOH	16.4 ± 10.2	0.0 ± 0.0
	Inula conyzae (Griess.) Meikle	Bo.	B	SZ0023	roots	PE	25.9 ± 5.6	3.0 ± 2.2
				SZ0024	roots	EtOAc	66.4 ± 8.3	5.4 ± 6.0
				SZ0025	roots	MeOH	21.2 ± 15.0	0.0 ± 0.0
				SZ0026	leaves + flowers	PE	3.3 ± 3.7	13.2 ± 18.5
				SZ0027	leaves + flowers	EtOAc	7.5 ± 3.9	0.0 ± 0.0
				SZ0028	leaves + flowers	MeOH	2.6 ± 3.7	10.3 ± 14.5
	Silybum marianum (L.) Gaertn.	–	A	P01490	aer. pts.	PE	11.4 ± 9.4	0.0 ± 0.0
				P01491	aer. pts.	EtOAc	19.8 ± 7.3	0.0 ± 0.0
				P01492	aer. pts.	MeOH	6.8 ± 7.1	0.0 ± 0.0
				P01493	fruits	PE	8.0 ± 5.1	11.0 ± 2.5
				P01494	fruits	EtOAc	24.7 ± 5.5	0.0 ± 0.0
				P01495	fruits	MeOH	23.9 ± 8.5	0.5 ± 0.8
Asteraceae	*Tanacetum parthenium* L.	Br., Lo., Zw.	A	P01511	aer. pts.	PE	21.9 ± 10.3	0.0 ± 0.0
				P01512	aer. pts.	EtOAc	37.6 ± 11.4	0.0 ± 0.0
				P01513	aer. pts.	MeOH	3.6 ± 5.1	0.4 ± 0.5
Brassicaceae	*Armoracia rusticana* G.Gaertn., B.Mey. & Scherb.	Bo., Lo, Ta.2, Zw.	A	P01445	roots	PE	9.4 ± 6.8	0.6 ± 0.9
				P01446	roots	EtOAc	29.8 ± 11.4	9.3 ± 3.8
				P01447	roots	MeOH	17.0 ± 4.5	2.1 ± 2.9
	Nasturtium officinale R. Br.	Zw.	A	P01602	aer. pts.	PE	26.1 ± 3.8	5.2 ± 5.0
				P01603	aer. pts.	EtOAc	7.3 ± 5.2	4.6 ± 3.8
				P01601	aer. pts.	MeOH	30.7 ± 12.7	8.5 ± 2.0
Cannabaceae	*Humulus lupulus* L.	Br., Lo., Ta.2	A	P01684	flowers	PE	31.3 ± 9.9	0.0 ± 0.0
				P01685	flowers	EtOAc	95.8 ± 2.6	4.0 ± 10.5
				P01686	flowers	MeOH	9.2 ± 5.6	3.1 ± 2.2
Caryophyllaceae	*Gypsophila muralis* L.	–	B	P01634	aer. pts.	PE	4.2 ± 3.5	0.0 ± 0.0
				P01635	aer. pts.	EtOAc	10.9 ± 8.1	8.5 ± 12.0
				P01636	aer. pts.	MeOH	4.7 ± 1.6	0.0 ± 0.0
Clusiaceae	*Hypericum perforatum* L.	Bo.,Fu., Lo., Ma., Ta.2	A	P01693	aer. pts.	PE	69.3 ± 17.2	32.3 ± 40.0
				P01694	aer. pts.	EtOAc	97.5 ± 1.2	20.0 ± 1.3
				P01695	aer. pts.	MeOH	16.8 ± 10.8	1.6 ± 2.3
Cucurbitaceae	*Bryonia alba* L.	–	A	P01487	roots	PE	18.0 ± 6.8	0.8 ± 0.6
				P01488	roots	EtOAc	13.7 ± 10.1	0.0 ± 0.0
				P01489	roots	MeOH	9.2 ± 5.0	0.0 ± 0.0
Ericaceae	*Arbutus unedo* L.	–	A	P01445	roots	PE	19.1 ± 7.8	0.0 ± 0.0
				P01446	roots	EtOAc	8.8 ± 10.4	0.7 ± 0.6
				P01447	roots	MeOH	4.4 ± 6.2	1.5 ± 2.1
Euphorbiaceae	*Euphorbia cyparissias* L.	–	A	P01572	aer. pts.	PE	11.0 ± 9.3	0.0 ± 0.0
				P01573	aer. pts.	EtOAc	18.4 ± 11.5	0.0 ± 0.0
				P01571	aer. pts.	MeOH	10.2 ± 7.3	0.0 ± 0.0
Fabaceae	*Anthyllis vulneraria* L.	–	A	P01439	flowers	PE	20.7 ± 5.0	13.5 ± 5.1
				P01440	flowers	EtOAc	20.8 ± 14.7	8.9 ± 5.8
				P01441	flowers	MeOH	15.8 ± 12.5	7.3 ± 3.7
	Robinia pseudoacacia L.	–	A	P01466	flowers	PE	26.7 ± 5.2	7.5 ± 1.8
				P01467	flowers	EtOAc	39.1 ± 5.6	8.0 ± 7.3
				P01468	flowers	MeOH	0.0 ± 0.0	0.0 ± 0.0
Gentianaceae	*Centaurium erythraea* Rafn.	Ma., Ta.1, Ta.2, Zw.	A	P01654	aer. pts.	PE	9.1 ± 6.5	5.4 ± 1.7
				P01655	aer. pts.	EtOAc	38.9 ± 4.2	0.0 ± 0.0
				P01656	aer. pts.	MeOH	3.6 ± 2.8	2.3 ± 3.2
	Gentiana lutea L.	Bo., Ma., Ta.2, Zw.	A	P01642	roots	PE	4.4 ± 2.1	7.9 ± 10.8
				P01643	roots	EtOAc	9.7 ± 5.4	0.0 ± 0.0
				P01644	roots	MeOH	0.3 ± 0.4	0.0 ± 0.0
Lamiaceae	*Galeopsis segetum* Neck.	–	A	P01578	aer. pts.	PE	18.1 ± 10.1	0.0 ± 0.0
				P01579	aer. pts.	EtOAc	7.6 ± 6.9	1.6 ± 2.2
				P01577	aer. pts.	MeOH	23.7 ± 7.6	2.4 ± 3.4

Tab. S1. (Cont).

Plant Family	Plant species	Historical source	Src.	Voucher specimen	Plant part	extract solvent	growth inhib. at 4.81 µg/mL ± SD[a]	growth inhib. at 0.81 µg/mL ± SD[a]
Lamiaceae	*Hyssopus officinalis* L.	Ma.	A	P01584	aer. pts.	PE	66.0 ± 8.3	0.0 ± 0.0
				P01585	aer. pts.	EtOAc	6.1 ± 4.3	1.8 ± 2.6
				P01582	aer. pts.	MeOH	64.1 ± 5.9	6.3 ± 5.3
	Nepeta cataria L.	Br.	A	P01605	aer. pts.	PE	35.2 ± 3.7	0.2 ± 0.3
				P01606	aer. pts.	EtOAc	5.8 ± 4.9	6.1 ± 8.7
				P01604	aer. pts.	MeOH	12.7 ± 2.4	0.0 ± 0.0
	Origanum dictamnus L.	–	A	P01542	aer. pts.	PE	16.4 ± 7.2	0.0 ± 0.0
				P01543	aer. pts.	EtOAc	33.1 ± 16.3	16.9 ± 4.8
				P01541	aer. pts.	MeOH	14.7 ± 7.6	3.9 ± 5.5
	Origanum vulgare L.	–	A	P01608	aer. pts.	PE	57.7 ± 15.0	5.3 ± 5.3
				P01607	aer. pts.	MeOH	53.3 ± 15.6	9.9 ± 9.4
	Stachys officinalis (L.) Trev.	Br.	A	P01478	aer. pts.	PE	30.5 ± 17.6	0.0 ± 0.0
				P01479	aer. pts.	EtOAc	30.5 ± 8.8	2.1 ± 1.5
				P01480	aer. pts.	MeOH	4.2 ± 5.8	0.0 ± 0.0
Piperaceae	*Piper cubeba* L.F.	–	A	P01520	fruits	PE	34.9 ± 2.4	18.3 ± 5.1
Polygonaceae	*Bistorta officinalis* Delarbre	–	A	P01481	aer. pts.	PE	7.1 ± 6.1	0.0 ± 0.0
				P01482	aer. pts.	EtOAc	23.3 ± 6.8	4.2 ± 3.0
				P01483	aer. pts.	MeOH	14.1 ± 5.7	0.0 ± 0.0
				P01484	roots	PE	11.1 ± 8.2	2.1 ± 2.3
				P01485	roots	EtOAc	12.6 ± 11.2	1.5 ± 2.1
				P01486	roots	MeOH	6.7 ± 7.5	0.0 ± 0.0
Ranunculaceae	*Aquilegia vulgaris* L.	–	A	P01442	aer. pts.	PE	18.1 ± 3.5	4.5 ± 4.0
				P01443	aer. pts.	EtOAc	42.7 ± 7.4	12.0 ± 0.8
				P01444	aer. pts.	MeOH	18.7 ± 2.3	5.7 ± 3.7
Rosaceae	*Alchemilla alpina* L.	–	A	P01533	aer. pts.	PE	67.3 ± 9.2	11.1 ± 3.2
				P01534	aer. pts.	EtOAc	12.6 ± 3.6	1.2 ± 1.7
				P01532	aer. pts.	MeOH	27.6 ± 5.4	10.6 ± 7.5
	Alchemilla vulgaris L. em. Fröhner	–	A	P01563	aer. pts.	PE	43.3 ± 2.6	5.8 ± 0.4
				P01564	aer. pts.	EtOAc	11.4 ± 0.6	3.1 ± 0.2
				P01562	aer. pts.	MeOH	11.1 ± 20.4	0.0 ± 0.0
	Agrimonia eupatoria L.	Br., Lo., Ma.,Ta.2, Zw.	A	P01699	aer. pts.	PE	47.9 ± 6.1	0.0 ± 0.0
				P01700	aer. pts.	EtOAc	20.4 ± 4.7	1.1 ± 1.1
				P01701	aer. pts.	MeOH	53.6 ± 17.6	21.8 ± 20.6
	Geum urbanum L.	–	A	P01505	roots	PE	8.6 ± 1.9	0.0 ± 0.0
				P01506	roots	EtOAc	14.7 ± 11.5	1.2 ± 1.7
				P01506	roots	MeOH	3.4 ± 3.1	0.0 ± 0.0
				P01507	aer. pts.	PE	11.0 ± 8.3	0.3 ± 0.4
				P01508	aer. pts.	EtOAc	25.0 ± 6.9	0.0 ± 0.0
				P01509	aer. pts.	MeOH	17.6 ± 8.1	3.6 ± 4.6
	Potentilla erecta (L.) Raeusch.	Br., Bo., Lo., Ta.2	A	P01696	roots	PE	7.6 ± 5.5	0.0 ± 0.0
				P01697	roots	EtOAc	23.8 ± 1.8	9.5 ± 3.8
				P01698	roots	MeOH	7.3 ± 1.9	1.8 ± 2.5
	Potentilla anserina L.	–	A	P01436	aer. pts.	PE	16.9 ± 12.3	7.7 ± 7.6
				P01437	aer. pts.	EtOAc	16.7 ± 15.9	18.9 ± 4.7
				P01438	aer. pts.	MeOH	24.4 ± 6.1	19.5 ± 8.9
	Potentilla aurea L.	Zw.	A	P01687	leaves	PE	11.1 ± 5.3	4.4 ± 4.9
				P01688	leaves	EtOAc	18.5 ± 1.6	6.1 ± 4.6
				P01689	leaves	MeOH	13.0 ± 6.3	5.3 ± 4.0
Rubiaceae	*Galium odoratum* (L.) Scop.	–	A	P01469	aer. pts.	PE	8.5 ± 9.7	0.0 ± 0.0
				P01470	aer. pts.	EtOAc	21.8 ± 4.5	0.0 ± 0.0
				P01471	aer. pts.	MeOH	20.4 ± 4.6	9.2 ± 13.1
Verbenaceae	*Verbena officinalis* L.	Bo., Ta.2, Zw.	B	P01690	aer. pts.	PE	8.8 ± 8.7	0.0 ± 0.0
				P01691	aer. pts.	EtOAc	40.9 ± 16.8	3.7 ± 3.5
				P01692	aer. pts.	MeOH	22.5 ± 19.0	1.5 ± 2.2

Source A: Plants were obtained from Dixa (St. Gallen, Switzerland).
Source B: Plants were collected in and around Basel by Dr. M. Adams in the summer of 2010.

Analytical methods

TLC

Thin layer chromatography plates (TLC silica gel 60 F254) were from Merck (Darmstadt, Germany). Mobile phase: ethyl acetate/*n*-heptane 30:70. Detection was done in a UV

chamber at 254 and 366 nm. Spots were also visualised with anis aldehyde-sulphuric acid reagent, which was prepared according to Wagner and Bladt [21].

HPLC ESI-MS

For micro fractionation and analysis of extracts an HPLC system consisting of a 1100 series low-pressure mixing pump with degasser module, column oven, and a 1100 series PDA detector (all Agilent, Waldbronn, Germany) was used. A Gilson 215 liquid handler with Gilson 819 injection module and 50 µl loop served as autosampler (Gilson; Mettmenstetten, Switzerland). The HPLC was coupled to an Esquire 3000 Plus ion trap mass spectrometer equipped with an electrospray (ESI) interface (Bruker Daltonics; Bremen, Germany). The MS parameters were as follows: Spectra were recorded under ion charge control conditions (ICCD 30 000) at a scan speed of 30 000 m/z/s with a Gauss filter with of 0.2 m/z. Nitrogen was used as a drying gas a flow rate of 10 L/min and as nebulising gas at a pressure of 30 psi. The nebulizer temperature was set 300 ° C. In the positive ion mode spectra were detected from 150–1500 m/z. Capillary voltage was set at -4500 V, endplate offset at -500 V. capillary exit at 109.8 V, skimmer voltage at 65.0 V, and trap drive at 39.8. The negative ion mode was also recorded from 150–1500 m/z. Capillary voltage was set at 4500 V, endplate offset at -500 V. capillary exit at -111.8 V, skimmer voltage at - 40 V, and trap drive at 43.7. A SunFire RP-18, 3.5 µm, 3 x 150 mm (Waters GmbH, Eschborn, Germany) was used for HPLC ESI-MS. A gradient consisting of A (H_2O + 0.1% formic acid) and B (acetonitrile + 0.1% formic acid) was used, starting at 90% A–10 % B and leading to 0% A–100% B in in 30 min, followed 100% B for 5 minutes. The flow rate was 0.5mL/min. Data acquisition and processing for HPLC system was performed using HyStar 3.0. software (Bruker Daltonics).

MPLC

A Büchi Sepacore system consisting of a control unit C-620, a fraction collector C-660, an UV photometer C-635, and two pump modules C-605 was used, with the following method. The column consisted of a cartridge (Büchi, ø 40 x150 mm) containing pressed silica gel (Silica gel 60, 0.040-0.063 mm, Merck, Darmstadt, Germany). A gradient system was used consisting of A (heptane) and B (ethyl acetate), starting at 100 % A and 0% B, and leading to 70% A and 30% B in 33 minutes, then to 20 % A and 80 % B in 31.5 minutes. The flow rate was 30 mL/min. Fractions were collected every 30 seconds. The sample was dissolved in A:B 1:1 at a concentration of 50 mg/mL and the injection volume was 10 ml.

Semi-preparative HPLC

Semi-preparative HPLC was done on an Agilent 1100 series HPLC system consisting of an 1100 series quaternary low-pressure mixing pump with degasser module, column oven, and a 1100 series PDA detector with a 1000 µL loop.) using a SunFire prep RP-18 column (5 µm, 10 x 150 mm, Waters GmbH, Eschborn, Germany). A gradient starting at 85% A (H_20 + 0.1% formic acid) and 15% B (acetonitrile + 0.1% formic acid) and leading to 40% A and 60% B in 15 minutes, then to 100 % B in another 5 minutes. Finally the column was flushed with 100% B for 7 minutes. The flow rate was 5 mL/min. The sample was dissolved in MeOH at a concentration of 50 mg/mL and the injection volume was 300 µl.

Preparative HPLC

Preparative HPLC was done on a SCL-10, HPLC system from Shimadzu (Kyoto, Japan).A SunFire™ prep C18 OBD™ (5 µm, 30x 150 mm, Waters, Ireland) was used. The gradient was isocratic for 30 min and consisted of acetonitrile:H_20 1: 1 at a flow rate of 30 ml/min. UV data were recorded from 220 to 500 nm. The samples were dissolved in acetonitrile at a concentration of 100 mg/ml and the injection volume was 300 µl.

High resolution Mass Spectrometry (micrOTOF)

High-resolution mass spectra were obtained on a micrOTOF ESI-MS system (Bruker Daltonics) connected to an Agilent 1100 series HPLC. Data acquisition and processing was performed using HyStar 3.0 software (Bruker Daltonics). Conditions for LC-TOF MS were as follows: spectra were recorded in the range of m/z 150–1500 in positive mode. Nitrogen was used as a nebulising gas at a pressure of 2.0 bar and as a drying gas at a flow rate of 9.0 L/min (dry gas temperature 240 °C). Capillary voltage was at 4500 V, endplate offset at -500 V, hexapole at 250.0 Vpp, skimmer 1 at −50 V and skimmer 2 at −22.5 V. Instrument calibration was performed using a reference solution of sodium formiate 0.1 % in isopropanol / water (1:1) containing 5 mM sodium hydroxide. Typical mass accuracy was ±2 ppm. The spectra were recorded in negative and positive mode in the range of m/z 150–1500.

NMR

NMR data were acquired at target temperature 18°C on a Bruker Avance III™ 500 MHz spectrometer (Bruker, Fällanden, Switzerland) operating at 500.13 MHz for ^{1}H, and 125.77 MHz for ^{13}C. A 1mm TXI microprobe with a z-gradient was used for ^{1}H-detected experiments; ^{13}C-NMR spectra were recorded with a 5 mm BBO probe head with z-gradient. NMR experiments were done as previously described [22]. For processing and evaluation Topspin 2.0 was used.

Bioassays

a. In vitro test against Trypanosoma brucei rhodesiense

Trypanosoma brucei rhodesiense (STIB 900) were grown in axenic medium as previously described [23].The compounds were tested using a modified Alamar Blue assay protocol [24] to determine the 50% inhibitory concentration (IC_{50}). Serial threefold drug dilutions were prepared in 96-well micro titer plates and 50 µl of *T. b. rhodesiense* STIB 900 bloodstream forms were added to each well except for the negative controls. Melarsoprol (Arsobal®, Sanofi-Aventis, Meyrin, Switzerland) was used as a reference drug. After 70 h of incubation Alamar blue marker (12.5 mg resazurin dissolved in 100 mL distilled water) was added. The plates were then incubated for an additional 2 to 5 h. A Spectramax Gemini XS micro plate fluorescence reader (Molecular Devices Cooperation, Sunnyvale, CA) with an excitation wavelength of 536 nm and an emission wavelength of 588 nm was used to read the plates. The IC_{50} values were calculated from the sigmoidal growth inhibition curves using Softmax Pro software (Molecular Devices).

b. In vitro testing against Plasmodium falciparum

A modification of the [^{3}H]-hypoxanthine incorporation assay was used to determine the intra-erythrocytic antiplasmodial activity (Des Jardins 1979) of the extract library and

purified compounds in 96 well plates. Chloroquine (Sigma-Aldrich) and artesunate (Mepha, Switzerland) were used as standard drugs. Briefly, infected human red blood cells in RPMI 1640 medium (100 µL per well with 2.5% haematocrit and 0.3% parasitaemia) were exposed to twofold serial drug dilutions in 96-well micro titer plates. After 48 h incubation, 0.5 µCi [^{3}H]-hypoxanthine was added to each well. The plates were incubated for further 24 h before being harvested using a Betaplate cell harvester (Wallac, Zürich, Switzerland) The radioactivity was counted with a Betaplate liquid scintillation counter (Wallac) as counts per minute per well at each drug concentration and compared to the untreated controls. IC_{50} values were calculated from sigmoidal inhibition curves using Microsoft Excel. All assays were run in duplicate and repeated three times [25].

c. In vitro cytotoxicity testing

Cytotoxicity was assessed using a similar Alamar Blue assay protocol [23] whereby 4000 rat myoblast cells/well were seeded in RPMI 1640 medium. All following steps were according to the *T. b. rhodesiense* protocol. Podophyllotoxin (Sigma-Aldrich) was used as the reference drug.

References

[21] Wagner H, Bladt S, editors.
Plant Drug Analysis. A thin layer Chromatography Atlas, 2th ed.
Berlin: Springer-Verlag, 1996: 359.

[22] Adams M, Plitzko I, Kaiser M, Brun R, Hamburger M.
HPLC-profiling for antiplasmodial compounds – 3-methoxy carpachromene from *Pistacia atlantica*.
Phytochem Lett. 2009; 2: 159–162.
http://dx.doi.org/10.1016/j.phytol.2009.05.006

[23] Baltz T, Baltz D, Giroud C, Crockett J.
Cultivation in a semidefined medium of animal infective forms of *Trypanosoma brucei*, *T. equiperdum*, *T. evansi*, *T. rhodesiense*, *T. gamiense*.
EMBO J. 1985; 4: 1273–1277.
http://www.ncbi.nlm.nih.gov/pubmed/4006919

[24] Räz B, Hen M, Grether-Bühler Y, Kaminsky R, Brun R.
The Alamar Blue assay to determine drug sensivity of African trypanosomes in vitro.
Acta Trop. 1997; 68: 139–147.
http://dx.doi.org/10.1016/S0001-706X(97)00079-X

[25] Desjardins RE, Canfield CJ, Haynes JD, Chulay JD.
Quantitative assessment of antimalarial activity *in vitro* by a semiautomated microdilution technique.
Antimicrob Agents Chemother. 1979; 16: 710–718.
http://dx.doi.org/10.1128/AAC.16.6.710
